# Experience of parents who have suffered a perinatal death in two Spanish hospitals: a qualitative study

**DOI:** 10.1186/s12884-019-2666-z

**Published:** 2019-12-19

**Authors:** Marcos Camacho-Ávila, Cayetano Fernández-Sola, Francisca Rosa Jiménez-López, José Granero-Molina, Isabel María Fernández-Medina, Laura Martínez-Artero, José Manuel Hernández-Padilla

**Affiliations:** 1 Gynaecology and Obstetrics Unit, Hospital de Torrevieja, Alicante, Spain; 2 Department of Nursing Science, Physiotherapy and Medicine, Universidad de Almería, Carretera de Sacramento, 04120 Almería, Spain; 3 Facultad de Ciencias de la Salud, Universidad Autónoma de Chile, Temuco, Chile; 4 Paediatric Unit, Hospital Torrecárdenas, Almería, Spain; 5 Gynaecology and Obstetrics Unit, Hospital de Poniente, El Ejido, Almería Spain; 6 Department of Adult, Child and Midwifery School of Health & Education, Middlesex University, London, UK

**Keywords:** Stillbirth, Perinatal death, Perinatal grief, Qualitative research

## Abstract

**Background:**

Perinatal grief is a process that affects families in biological, psychological, social and spiritual terms. It is estimated that every year there are 2.7 million perinatal deaths worldwide and 4.43 deaths for every 1000 births in Spain. The aim of this study is to describe and understand the experiences and perceptions of parents who have suffered a perinatal death.

**Methods:**

A qualitative study based on Gadamer’s hermeneutic phenomenology. The study was conducted in two hospitals in the South of Spain. Thirteen mothers and eight fathers who had suffered a perinatal death in the 5 years prior to the study participated in this study. In-depth interviews were carried out for data collection. Inductive analysis was used to find themes based on the data.

**Results:**

Eight sub-themes emerged, and they were grouped into three main themes: ‘Perceiving the threat and anticipating the baby’s death: “*Something is going wrong in my pregnancy”*’; ‘Emotional outpouring: the shock of losing a baby and the pain of giving birth to a stillborn baby’; *“We have had a baby”*: The need to give an identity to the baby and legitimise grief’.

**Conclusion:**

The grief suffered after a perinatal death begins with the anticipation of the death, which relates to the mother’s medical history, symptoms and premonitions. The confirmation of the death leads to emotional shock, characterised by pain and suffering. The chance to take part in mourning rituals and give the baby the identity of a deceased baby may help in the grieving and bereavement process.

Having empathy for the parents and notifying them of the death straightaway can help ease the pain. Midwives can help in the grieving process by facilitating the farewell rituals, accompanying the family, helping in honouring the memory of the baby, and supporting parents in giving the deceased infant an identity that makes them a family member.

## Background

According to the World Health Organisation (WHO), perinatal death is the death of a baby between 22 weeks of gestation (or weighing 500 g) and 7 days after birth [1]. Although perinatal mortality has gone down globally [2], there were 2.6 million perinatal deaths in 2017 [3]. In Europe the perinatal mortality rate was between 4 and 6/1000 [4]. In Spain it has decreased from 20 deaths for every 1000 births in 1975 to 4.20 deaths for every 1000 births in 2018 [5].

Perinatal grief includes biological, psychological, social [6] and spiritual factors [7] During this process, the parents may suffer eating and sleeping disorders [8], more chronic diseases, and a lower quality of life [9]. They also suffer anxiety, depression [10, 11] post-traumatic stress disorder [12–14], and many other mental health issues [15–18].

This type of death produces social changes on a family level [19]. Relationships with the older children are affected [20–22] because the parents may become overprotective [23] of them or even distant [24]. Children experience feelings of guilt, fear and anxiety [25], which puts a strain on the parents’ relationship and increases conflicts between them [15, 26].

Perinatal grief is framed within a social context in which a perinatal death is not recognised as the death of a baby [27–29]. Research shows that after a perinatal death, parents receive inaccurate information too late [30, 31] and receive inappropriate comments from healthcare professionals [32]. In Spain, unlike in other countries, there is no standardised care for families suffering perinatal grief, with a great variability of care in daily practice for mothers and fathers who suffer the death of their baby [33]. Some studies have shown that in Spain many parents whose babies died did not have a chance to say goodbye to them and have no memory of them such as a photograph or fingerprints as they were never offered these possibilities [34].

Worden’s model defines grief as an adaptation in which “tasks” must be completed by the person who suffers a loss. These “tasks” are: to accept the reality of the loss; work through the pain of the baby’s death; adjust to an environment in which the deceased baby is missing; and find an enduring connection with the deceased baby while embarking on a new life [35]. This way of approaching grief gives those suffering a loss an active role in their mourning [36].

Literature on the subject shows both a scarcity of clinical guidelines [37] and the need for more evidence on the phenomenon from the perspective of the mothers and fathers of the deceased baby [38].

## Methods

### Aim

The objective of this study is to describe and understand the experiences and perceptions of mothers and fathers who have suffered a perinatal death.

### Design and setting

A qualitative study based on Gadamer’s hermeneutic phenomenology was designed. For Gadamer, human experience cannot be understood without language [39]. Understanding participants’ stories requires being prepared to be told something through a dialogue, from which meanings emerge. The development of the study followed the phases of a Gadamerian-based research method [40]:

1. To decide whether the research question is pertinent to the methodological assumptions. Perinatal loss is a phenomenon of the lifeworld that can be understood from the perspective of hermeneutic phenomenology. Gadamer’s philosophical approach is appropriate for comprehending the experiences of parents who have suffered a perinatal death.
2. To identify the researchers’ pre-understanding of the topic (Reflexivity). The pre-understanding of the researchers came from their clinical experience as healthcare professionals who work or have worked in a delivery room as midwives.

The study was carried out in Torrevieja Hospital and Vinalopó Hospital, in Alicante, Spain. They are both public hospitals and have an average of 1400 births per year.

### Population and participants

A convenience sample of parents who had suffered a perinatal death was recruited. The histories of perinatal deaths in both hospitals over the last 5 years was consulted. A total of 63 perinatal deaths took place in the hospitals in the 5 years prior to the study. Mothers and fathers who fulfilled the inclusion criteria were called, and the aim of the study was explained to them. They were told that their participation was voluntary and they could choose not to reply or abandon the study at any time. It was explained that the data obtained would be treated confidentially and in accordance with European data protection regulations. The inclusion criteria were: (1) Being a mother or father who has suffered a loss through the perinatal death of their child, from the 22nd week of gestation to the first week of life. (2) The death occurring between 3 months and 5 years prior to the study was decided on as the memory of a very recent loss could be very painful. This would provide a broad sample and ensure that the experience would be remembered with sufficient detail and intensity. (3) The mother or father had to speak Spanish or English. (4) Signing the informed consent form.

A total of 13 mothers and 8 fathers were interviewed (see Table 1). Of the 63 cases of perinatal death registered in this period, eleven did not answer the telephone, eight did not speak Spanish or English, nine refused to discuss the subject, and nine claimed they did not have time to be interviewed.

**Table 1:** Socio-demographic data of the sample

Participant	Nationality	Education	Employment	Time of death	Baby’s age	Time since death of baby	Place of interview
P-1	Colombian	Secondary	Employed	Intrapartum	40 weeks	12 months	Health Centre
P-2	Colombian	Secondary	Unemployed	Antepartum	30 weeks	36 months	Health Centre
P-3	Spanish	Secondary	Employed	Intrapartum	24 weeks	48 months	Health Centre
P-4	Spanish	University	Employed	Intrapartum	24 weeks	48 months	Health Centre
P-5	Spanish	Secondary	Employed	Antepartum	34 weeks	60 months	Health Centre
P-6	Spanish	University	Employed	Postpartum	6 days	5 months	Health Centre
P-7	Spanish	University	Employed	Postpartum	6 days	5 months	Health Centre
P-8	Spanish	Primary	Employed	Postpartum	3 days	18 months	Home
P-9	Spanish	Primary	Employed	Postpartum	3 days	18 months	Home
P-10	Ecuadorian	Secondary	Unemployed	Antepartum	28 weeks	24 months	Health Centre
P-11	Spanish	Primary	Employed	Antepartum	40 weeks	18 months	Home
P-12	Spanish	Primary	Unemployed	Antepartum	40 weeks	18 months	Home
P-13	Spanish	Secondary	Employed	Intrapartum	24 weeks	30 months	Health Centre
P-14	Spanish	University	Employed	Antepartum	36 weeks	6 months	Health Centre
P-15	Spanish	Primary	Employed	Antepartum	34 weeks	24 months	Health Centre
P-16	Spanish	Secondary	Unemployed	Antepartum	38 weeks	36 months	Home
P-17	Spanish	Primary	Employed	Antepartum	38 weeks	36 months	Home
P-18	Spanish	Primary	Employed	Antepartum	37 weeks	3 months	Hospital
P-19	Spanish	Secondary	Employed	Antepartum	37 weeks	3 months	Hospital
P-20	Spanish	Secondary	Employed	Antepartum	38 weeks	36 months	Health Centre
P-21	Spanish	University	Employed	Antepartum	25 weeks	15 months	Hospital

Age range: 26–43 years old. Standard deviation: 4.76 years

### Data collection

Open, in-depth interviews were used for data collection. The interviews took place between April 2016 and May 2017. The parents were contacted and invited to take part by the main researcher. The interviews were carried out by two researchers who were midwives, and one who was a paediatric nurse. Each interview lasted between 45 and 60 min, and they were audio recorded. The interview started with an open-ended, general question: “What was the experience of losing your baby like?” Subsequently, other questions were asked following the protocol used and based on the natural flow of the conversation. The final question in each interview was: “Do you have anything else you would like to add?” The interviewer also took note of non-verbal signs throughout the interview. During the interviews, participants were reminded of ethical issues and were told that recounting their experience could be helpful in improving care for other parents in their same situation, times were respected, and the emotional and psychological needs of the mothers and fathers were met, for example, by pausing or letting them express their emotions. When the researchers perceived that no new issues were emerging and the same topics were being repeated, it was considered that the saturation of the data had been reached, and the data collection was concluded.

### Data analysis

The following steps were followed for the data analysis [40]:

1. To achieve understanding of the topic through dialogue with the participants. During the interviews, a spontaneous clarification regarding what the participants discussed was achieved by using follow-up questions (e.g., “Could you tell me what you mean when you say that the gynaecologist had a cold manner?”)
2. To conduct a conversation between the researchers and the participants through the text. Each transcription was analysed line by line in order to identify meaningful and important phrases and select them as quotes. Each quote was assigned a code that captured its meaning, grouped into units of meaning, sub-themes and themes. Data coding was performed individually by three researchers. They then compared their interpretations so each unit of meaning, theme and subtheme were agreed upon by consensus.

### Rigour

In order to ensure the rigour of the study, the participants were given the opportunity to confirm the transcriptions, units of meaning, sub-themes and themes by reading their answers. Additionally, all the participants’ experiences were represented. The study’s credibility was complemented by the triangulation of the researchers.

## Results

Eight sub-themes emerged, and they were grouped into three themes that help us to understand the experience of parents who have suffered a perinatal loss (Table 2).

**Table 2:** Summary of themes, sub-themes and units of meaning

Theme	Subtheme	Units of meaning
Perceiving the threat and anticipating the death: “Something is wrong with my pregnancy”	“This could end badly.” Medical history as a threat and a source of uncertainty	Medical history, infertility treatment, high-risk pregnancy, repeated miscarriage, vulnerability of the pregnancy, frequenting emergency services, suspicion
	Anticipating the death. From suspicion to confirmation	Warning signs, having a hunch, lack of movement, decreased movement, contractions, pain, worry, fear, helplessness
Emotional outpouring: the shock of losing a baby and the pain of giving birth to a stillborn baby	Emotional shock upon notification of the baby’s death	Notification, non-verbal language, silence, serious expression, scarce explanation, hopelessness, disbelief, anguish, anger, emptiness, insurmountable pain.
	Giving birth to a stillborn baby: a doubly painful labour process for families	Caesarean, inducing labour, vaginal birth, extra suffering, not seeing the stillborn baby, anger about disregard from professionals, reassuring, professionalism
	Loneliness and lack of information as aggravating factors in the pain of the loss	Receiving the news alone, lack of information, unclear diagnosis, knowing the reason, demanding information, alleviating the pain, overcoming feelings of guilt
“We have had a baby.” The need to give the baby an identity and legitimacy to the grief	Saying goodbye to the deceased baby, having the baby’s footprint, keeping the memory of the baby alive	Seeing the deceased baby, embracing the baby, having photographs, keeping a footprint, saying final goodbyes, need for identification, need for recognition as a part of the family
	Mourning rituals. The importance of respecting individual beliefs	Baptism, cremation, burial, spiritual suffering, non-recognition, refusing baptism, keeping the ashes, having a meaningful place to visit the deceased, remembering the experience.
	Bureaucracy and administrative language as obstacles in the mourning process.	Administrative slowness, misinformation about administrative processes, inappropriate language, referring to the baby as a foetus, denying registry, denial of the existence, identification as a deceased baby.

### Theme 1. Perceiving the threat and anticipating the death: “Something is wrong with my pregnancy”

Mothers in the study often had a hunch that something was going wrong with their pregnancy. This feeling might have been related to the difficult process of assisted reproduction, a medical history of miscarriages, a high-risk pregnancy, or noticing a decrease in foetal movements. When a perinatal death is sensed in this way, mothers start assimilating the death right away.

*“… I was worried, I got the feeling that I was exaggerating it too much (…), but then I started to assimilate the fact that it would end badly”* (P-10).

#### “It could end badly”. Medical history as a threat and a source of uncertainty

Some parents have a long history of infertility treatments or miscarriages. According to the participants, pregnancies achieved through rigorous clinical procedures can leave an emotional footprint on couples, increasing their fears. Having such a background seemed to help parents to quickly assume the possibility of a loss and usually make them more aware of the fragility of the pregnancy.

*“I had been trying to have children for six years straight. My husband and I would get up at 2:00 a.m. for the treatments, (…) it was very stressful and I felt physical and psychological fatigue. After that, you’re always worried that something’s not right”* (P-5)

Some of the pregnancies were considered high-risk because of pre-existing conditions in the mothers such as hypertension or diabetes. These conditions made parents experience the pregnancy in a distressing way. The parents reported frequent visits to emergency services upon noticing any minor issue and having requested follow-up ultrasound scans in order to ease their feelings of uncertainty.

*“The doctors diagnosed me with a high-risk pregnancy (…). I had to get an ultrasound every month, but on top of that, I often went to the hospital, for aches, pains, bleeding (…). I was afraid something would happen”* (P-12).

#### Anticipating the death. From suspicion to confirmation

The warning signs of a perinatal death consist mainly of changes in the foetus’ movement patterns (from a decrease to a complete absence of movement). This change in movement patterns caused great uncertainty for the mothers that took part in the study.

*“(…) it had been a few days since I had felt any movements (…)*. *Since I already had an appointment for foetal monitoring, I didn’t want to go to the emergency room in case I was overreacting, I didn’t want to bother them although I was really worried”* (P-14).

Some mothers recalled pain and periodic or isolated contractions that they associated with cramping. Sometimes, these contractions led to active labour. In such cases, the physical pain of labour was compounded by the emotional pain and the feeling of helplessness when losing a baby and not being able to do anything to stop it.

*“The cramps that I felt (…) weren’t periodic or regular. I had some cramping, later a little more, then they told me that I had been dilating and my cervix had effaced (…) At that point, there was choice but to go ahead, and that made it even harder, if that’s even possible”* (P-13).

### Theme 2. Emotional outpouring: the shock of a baby’s death and the pain of giving birth to a stillborn baby

The confirmation of a baby’s death is the start of a long, hard journey that makes up part of the parents’ grief process. The emotional outpouring is produced once the death has been confirmed. The expectations the parents had for their baby are no longer present, and the pain of the loss increases due to uncertainty or loneliness. There were cases in which the babies were born alive and lived for a few hours or days, which resulted in even stronger attachments as well as more intense shock over the subsequent death of the baby.

*“You feel terrible, devastated, you go in there thinking you’re going to be a father, and suddenly your baby is dead”* (P-11).

#### Emotional shock upon notification of the baby’s death

Physicians often inform the parents whose babies passed away in critical care units. Parents recalled a strong emotional shock because, despite the warnings of a high-risk situation, they still hoped that they would be able to take their healthy baby home.

*“ The doctors told me that the next few hours were really important in his development (…) they called us on the phone, and of course, my heart started racing. They told us to come quickly because the baby was worse (…)*, *your world falls apart, and you lose all hope”* (P-10).

Parents recalled that they felt disbelief, anguish, anger, emptiness, insurmountable pain, and outrage about the loss of their baby. This represents the first step in working through their grief.

*“I didn’t know what to do, where to go, if I should just run away, hit something, scream, or do something. You are just stuck there in shock (…) you feel such intense pain yet emptiness at the same time (…) God! You can’t begin to imagine what it feels like”.* (P-11).

If the baby’s death occurs before birth, the notification of the death can be an extremely delicate situation. The participating parents recounted how they felt when doctors confirmed there was no foetal heartbeat.

*“I think the gynaecologist should have waited until my husband was there and have given us the news in a different way, but she started to check me with the sonogram, she looked at me and said: ‘No, I’m sorry, she doesn’t have any vital signs, she’s dead.’ Just like that “* (P-2).

According to the parents in the study, non-verbal language can be vital when receiving this devastating news: silence and the facial expressions of the gynaecologists and midwives can tell much. The delay in providing information and the scarcity of explanations also increased parents’ suffering.

*“The gynaecologist made a strange face, and I said, “Something’s wrong, isn’t it? (…) Please tell me everything’s ok.” She didn’t say anything, but her face said it all.”* (P-6).

In some cases, doctors consulted with a colleague before speaking to parents, which increases the waiting time and causes anxiety, tension, and uncertainty.

*“I could tell they were not saying anything to me during the ultrasound, then they called the other doctor in, and I got really nervous. And when I saw their faces, I asked, “What is it, is he dead?” because they weren’t telling me anything”* (P-2).

#### Giving birth to a stillborn baby: a doubly painful labour process for parents

When the death occurred before birth, the pain of the loss was increased by the physical labour pain of giving birth to a stillborn baby. In all cases, the mothers and fathers were advised to try a vaginal birth. One father asked for the caesarean section to avoid an even more difficult situation.

*“I didn’t want to see my son born dead, … it broke my heart (…*) *My wife and I thought that it would just draw out the situation and create unnecessary suffering”* (P-18).

The feeling of losing the baby, the pain of childbirth, and the fact that midwives may not be immediately available lead to feelings of anger and neglect in some parents.

*“(...) The pain was unbearable. I had called several times, and no one came until I finally went out into the hallway and yelled to get the midwife. The nurse’s aide told me that he was busy, and I responded angrily: “I don’t care where he is, but where he should be is in this room with my wife!”.* (P-21).

During such an unpleasant and emotional situation, receiving individualised care from midwives and physicians could become the most important source of comfort for parents.

*“I was impressed by the delivery room midwife, … the warmth with which she treated my wife. She let me stay with her, she held her hand and spoke to her gently … at such a hard time, that sort of personal treatment was comforting, and even today we still remember it as the most positive thing about that sad experience”.* (P-21).

#### Loneliness and lack of information as aggravating factors in the pain of the loss

The participating parents described various elements that made the grief process even harder. Some mothers were alone at the time they were notified about the death as their partners were not allowed to enter the ultrasound room.

*“Getting the news without having my husband there, you feel helpless and alone, (…) think about if someone told you something like that without anyone there, even to put their hand on your shoulder to console you, it’s shocking, right? It’s just wrong”.* (P-12).

Even when surrounded by many people, mothers sometimes felt just as lonely as they did when they were physically alone. As one mother pointed out, making parents who have lost a baby share the same hospital room with other parents that have given birth to a healthy baby increases suffering.

*“(…) I wasn’t in the mood to be there, in the same room where you can hear newborn babies in their cribs, and bottle carts go by, it was frustrating.”* (P-3).

Another element that added to the suffering was not knowing the cause of death or not having a clear explanation about the causes.

*“They should tell you things as they really are* [with emphasis]*. (…) But no, instead they tell me that my baby was born tired (…)* [Pause]. *This is the story the paediatricians told me, that my child was born tired. How can you say that, in that way?”* (P-1).

The results of the autopsy usually take a long time to come out. This prolonged uncertainty and prevented parents from achieving closure and moving on to the process of readapting to the world without their deceased baby.

*“On top of that, they make you wait months to get the autopsy back, and waiting such a long time with that same anguish and uncertainty doesn’t let you really live or move on.” (P-6).*

On most occasions, the autopsy is inconclusive and does not clarify the cause of death. This deepens parents’ suffering and prevents them from getting over feelings of guilt. Parents search for answers to questions such as: *What did I do wrong? Why didn’t I go to the doctor sooner?* The parents demand more information to understand the reasons behind what happened in the hope of finding relief for their pain and getting rid of their feelings of guilt.

*“He told me, “Everything was fine.” And I said, “What do you mean, everything was fine?” (…) That’s the explanation I was given (…) Can you believe that? All I wanted to know was the reason why and that I wasn’t to blame.”* (P-14).

*“I had been waiting for that appointment with the gynaecologist for such a long time..., to continue without answers. It would have helped me to understand why that had happened to me”* (P-15).

### Theme 3. “We have had a baby”: the need to give the baby an identity and legitimise grief

This theme reflects the parents’ need to give their deceased baby an identity as a member of the family and recognise their grief process. Parents valued having the opportunity to say farewell, keep memories of their baby and participate in mourning rituals based on their beliefs.

#### Saying goodbye to the deceased baby, having the baby’s footprint, keeping the memory of the baby alive

The participating parents valued the gynaecologists’ and midwives’ efforts to help them to say goodbye to the baby: allowing physical contact, keeping the umbilical cord clamp and the baby’s clothes or foot/handprints. In line with our theoretical framework, this eases the task of establishing an enduring connection with the deceased baby.

*“We appreciate the fact that we were allowed to be with him, see him, touch him (…) it was very hard, but we had to say our last goodbyes because even though he wasn’t born alive, he was still our son, and he would be forever.”* (P-11).

*“There are people that think that me having pictures of my son on my phone is gruesome. I don’t sit there looking at the photo all day, but if I need to, I know I can look at it, and that helps”* (P-5).

Some parents got distressed because some midwives did not offer them the possibility of keeping some mementos of their babies and recommended not seeing the baby. Not having time to say goodbye leads to suffering.

*“My daughter was beautiful and (…) they wrapped her up and took her away immediately, they didn’t let me see her or hold her at all (…) and I told them, “Wait!” and they didn’t wait. They told me it wasn’t good for me to spend a long time with her.”* (P-17).

#### Mourning rituals. The importance of respecting individual beliefs

Regarding post-mortem rituals, participating mothers and fathers agreed on the need for each set of parents to be able to act according to their beliefs. The possibility of baptising babies was something that was highly valued among Christian families as it made the process more bearable. However, when the gynaecologists, midwives and priests refused this possibility, the parents suffered because of the lack of legitimate recognition of their babies.

*“Couldn’t they have offered us the possibility of baptising her?...What fault did the baby have that she had left this world so soon, so little? The priest told us that we couldn’t have a mass for her, it’s not fair that he wouldn’t do anything for her, because she was and will continue to be my child.”* (P-4).

Such rituals, as well as choosing a final resting place can help parents to make a long-lasting connection with their babies. It is important that all parents decide how to proceed with the corpse according to their beliefs and needs.

*“I picked up the ashes, and that was it, and he’s with me. In the summer, I go to a different house, and I take my puppies and my son’s ashes (...) it’s just something that I need (…) to know that he’s there.”* (P-19).

*“Every week or fortnight, I go up to the cemetery, nobody can take away those five minutes I have with her. (…) Being able to go up there and be with my daughter puts my mind at ease.”* (P- 8).

Others parents did not worry about the religious rituals or the final resting place of the baby. Especially in the case of gestational stillborn children, where the hospital took care of the foetus. They felt it re-created the pain, and they avoided the topic completely.

*“That (stillbirth) was a painful experience, and I don’t need to have the remains of my baby to remember her. I remember the experience, but the baby no, because I didn’t get to meet her, they didn’t give us her body. My wife thinks about that more than I do, but neither of us wishes we had had a ceremony or had kept her remains”* (P-21).

#### Bureaucracy and administrative language as obstacles in the mourning process

The majority of parents recalled the administrative proceedings after the death to be a slow process. They express that greater diligence and better information on the steps to follow would have made this situation easier.

*“They should tell you what to do when your baby died, that you have to go to the funeral parlour, what papers they’re going to ask you for. They should direct you and guide you … “* (P-6).

The parents call for more appropriate language when referring to their deceased baby during the administrative processes. Referring to the baby as a “foetus” is considered derogatory and generates pain and suffering.

*“There were a few things that we didn’t like … for example, (in the report) it said ‘foetus’. That was pretty painful. No, for us, it’s not a foetus (…) it’s our baby”* (P- 20).

Furthermore, if the baby was not born alive, parents could not register him/her in the civil registry as a member of the family unit. This takes away the baby’s identity and reduces the legitimacy of the grief.

*“Since he wasn’t alive for one minute, neither his birth nor death could be registered*, *and I just broke down. When I went to the registry, I started crying (…) it’s as if my son didn’t exist”* (P-17).

## Discussion

The objective of this study was to describe and understand the experiences and feelings of mothers and fathers who have suffered a perinatal death. The study findings suggest that some parents perceive “there is something wrong with the pregnancy” and anticipate the foetus loss. This could be influenced by either previous experiences or physical signs that make them suspect. The baby’s death triggers an emotional outbreak characterized by shock, pain and sharpened by the feeling of loneliness. Parents need to legitimize grief, giving identity to the dead baby, saying goodbye to him and participating in rituals according to their beliefs.

Parents start thinking about a possible perinatal death when they notice a decrease or absence of foetal movement [41–43]. However, some women simply had a feeling or a hunch, which may be explained by the strong physical, emotional and spiritual connection between a mother and her baby [44–46]. The study conducted by Erlandsson et al. [47] points out the importance of educating mothers on typical intrauterine movement patterns and the gut feelings that a mother gets when she knows something is wrong [31]. These suspicions have been interpreted as a predictor for a death, which precedes the acceptance of such [34].

The majority of intrauterine deaths recounted in our study were diagnosed using ultrasound, which the participating mothers and fathers remember as an agonising and sad process [42]. Parents feel that the process of being informed takes too long, and they tend to assume something is wrong as soon as they see the non-verbal language of healthcare professionals conducting the ultrasound [48, 49]. Research suggests that notifying the death in a clear manner is best, taking care to avoid patronising parents and maintain respect for the individual’s preferences [50–52].

Consistent with other studies, the response of mothers and fathers to a perinatal death is characterised by feelings of anger, shock, disbelief, denial, despair and hopelessness [53, 54]. These feelings are stronger when women were not accompanied by their partners at the time of receiving the news of the death. In these cases, feelings of fear, anguish, and dissatisfaction with the care arise [55]. The results point to the need to restore the role of the husband, whose pain during a perinatal death is often relegated to a secondary role by society and healthcare professionals [56].

Our participants found any information regarding the cause of death to be useful, helping them to free themselves from feelings of guilt. However, they often do not find out what the actual cause was [57, 58]. In these cases, literature on the topic underscores the importance of healthcare professionals’ support [59].

Certain practices that ease pain amongst the mothers in our study coincide with those described in other studies; these practises are: seeing the baby; making time to say goodbye, and keeping mementos such as photographs, footprints or clothing [60, 61]. Participants agree that being encouraged to perform mourning rituals and keeping memories of their baby is a positive idea [9, 62]. In the case of parents not wishing to do so, healthcare professionals should respect their decision [61]. Furthermore, healthcare professionals should encourage parents’ intimacy and avoid placing them in the maternity area as if they were parents of healthy babies [12, 63, 64].

The absence of care protocols can lead to the refusal or opposition to farewell rituals [17]. Consistent with other studies [65–67], the need to give the deceased baby an identity as a member of the family is clear. Referring to the baby as a foetus in the medical reports and denying parents the possibility of registering the baby in civil registries contributes to parents’ frustration and “disenfranchised grief” [28].

One of the study’s limitations is in its sample selection. The participants recruited for this study had diverse life experiences; some of them had had experiences of miscarriages or infertility whilst others had not. This could have influenced our results, particularly in theme 1. Concerning the data collection, despite having performed individual interviews and reaching data saturation, using focus groups would have contributed to the interaction of the participating mothers and fathers and could have enriched the results. Another limitation is the lower number of men in the sample since they refused more to talk about the subject or were busy with work. A third limitation was the inability to interview non-Spanish and English-speaking mothers and fathers as there were quite a few parents with other languages such as Arabic, Russian or Romanian who could not be interviewed due to the language barrier.

## Conclusions

The findings of this study show that parents who face a perinatal loss tend to anticipate their baby’s death as the often perceive that “something was wrong with their pregnancy”. This study also shows that the shock of losing a baby and the pain of giving birth to a stillborn baby triggers an emotional outpouring in parents, who feel that they “have had a baby” and need to give him an identity to legitimate the grief.

The participating parents have had suspicions, based on their clinical background and anticipated the death of their children before the signs of alarm and foreboding. The participating fathers and mothers report an emotional shock at the death of their baby, which, in the case of antepartum death, is increased by the physical and emotional pain of giving birth to a dead child.

Loneliness, lack of empathy and information deepened the feeling of pain from the death of the baby. Parents express the need to give identity to their dead baby and legitimacy to their grieving process. Saying goodbye to the baby and carrying out rituals according to the beliefs of each person favours the grieving process, but the bureaucracy and the clinical-administrative language (of the reports) worsen the feeling of pain.

For future research, taking into account the social and family implications that affect perinatal grief, it would be of interest to conduct interviews with family and friends of those who have suffered a perinatal death. This could contribute to understanding the character of this “disenfranchised grief” [27], which participants in this study describe.

## Data Availability

The datasets generated and analysed during the current study are not publicly available due technical issues. Data is based on recorded interviews and their transcriptions are only available for ATLAS.ti software, but are available from the corresponding author on reasonable request.

## Abbreviations

P: Participant
WHO: World Health Organization
